# Functional Masticatory Angle and Hyoid Bone Position: A Pilot Study on Occlusal Symmetry and Morphofunctional Adaptation

**DOI:** 10.3390/dj13100451

**Published:** 2025-10-01

**Authors:** Lorena Sigwald-Serpa, Icíar Sanz-Orrio Soler, Laura Marqués-Martínez, Juan-Ignacio Aura-Tormos, Esther García-Miralles, Clara Guinot-Barona

**Affiliations:** 1 Faculty of Medicine and Health Sciences, Catholic University of Valencia, San Vicente Martir, 46001 Valencia, Spainiciar.sanz@ucv.es (I.S.-O.S.); clara.guinot@ucv.es (C.G.-B.); 2 Dentistry Department, Faculty of Medicine and Dentistry, University of Valencia, 46010 Valencia, Spain; juan.aura@uv.es (J.-I.A.-T.); m.esther.garcia@uv.es (E.G.-M.)

**Keywords:** mastication, unilateral mastication, dental occlusion, hyoid bone, functional masticatory angle, planas technique

## Abstract

**Background**: The hyoid bone is a key anatomical structure involved in the functional coordination of the stomatognathic system. Although its position may vary in response to masticatory patterns, its relationship with functional occlusion remains insufficiently studied in orthodontics. **Objective**: This pilot study aimed to explore the association between masticatory type and hyoid bone position and to assess the clinical utility of the Functional Masticatory Angle of Planas (AFMP) in classifying masticatory patterns. **Materials and Methods**: A descriptive, observational, cross-sectional study was conducted with 18 patients. Right and left AFMPs were measured using standardized intraoral photographs, and hyoid bone position was assessed via panoramic radiographs, classified as either aligned or displaced. Measurements were repeated to assess intraobserver reliability. **Results**: In most cases, hyoid bone elevation occurred on the same side as the smaller AFMP, suggesting a possible adaptive response to unilateral masticatory dominance. High intraobserver agreement was confirmed for both AFMP and hyoid measurements. **Conclusions**: The findings suggest a potential relationship between functional masticatory asymmetry and hyoid bone position. While further studies with larger samples are needed, the AFMP appears to be a promising tool for evaluating functional occlusion in relation to craniofacial dynamics.

## 1. Introduction

The stomatognathic system is a functional unit involving bones, muscles, ligaments, joints, and teeth, coordinating essential functions such as mastication, swallowing, and phonation [1,2,3,4,5]. This system develops from early infancy, where functional stimuli such as breastfeeding contribute to mandibular growth and neuromuscular maturation. According to the Laws of Planas, alternating bilateral mastication ensures balanced maxillofacial development, while unilateral mastication may predispose to asymmetries in occlusion, posture, and craniofacial morphology [2,6].

The hyoid bone plays a pivotal role in mastication, deglutition, phonation, and head posture [7,8,9]. Its unique anatomical position, suspended without direct bony articulation, makes it a dynamic structure influenced by supra- and infrahyoid muscle activity. Morphological variations in size and degree of fusion of the cornua may affect its biomechanical role [8,10].

Developmentally, the hyoid originates from the second and third pharyngeal arches, with ossification patterns continuing postnatally [8,10]. Topographically, it serves as a key anatomical reference within the anterior cervical triangle, contributing to the boundaries of submandibular, carotid, muscular, and submental subtriangles [10].

The hyoid’s position is influenced by muscular activity, growth, and posture. Extension movements of the head affect hyoid alignment due to muscular traction, potentially altering mandibular development and facial patterning [8,11]. Dysfunction of the suprahyoid and infrahyoid musculature may lead to an abnormal descent of the hyoid, which can obstruct airflow and contribute to respiratory conditions such as obstructive sleep apnea [8,11]. Moreover, postural imbalances—including forward head posture and cervical muscle asymmetry—have been associated with mandibular deviations and malocclusion, supporting a bidirectional interaction between occlusion, posture, and craniofacial growth [8].

Masticatory and accessory muscles, including the masseter, temporalis, pterygoids, digastric, geniohyoid, mylohyoid, and sternohyoid, coordinate mandibular movements and stabilize the hyoid [1,10,12]. These muscle groups interact through coordinated elevation, depression, and anterior–posterior displacement of the hyoid, allowing efficient oral functions. The hyoid serves as a muscular anchor and mediates forces between the mandible, skull base, and cervical spine.

Various factors such as malocclusion, parafunctions, and trauma can disrupt masticatory function, potentially exceeding the system’s adaptive capacity and leading to functional decompensation with clinical manifestations [1,6]. Adaptive mechanisms vary by age, with younger individuals exhibiting modeling and remodeling, while adults depend mainly on remodeling processes [1]. Biomechanically, mandibular movement involves coordinated rotational and translational actions across the temporomandibular joints, enabling complex three-dimensional displacements [3]. Within this context, temporomandibular disorders are highly prevalent worldwide, affecting roughly one-third of the population, with higher rates in women and in younger individuals [13].

The hyoid bone supports swallowing, phonation, and postural balance through coordinated suprahyoid muscle activity, contributing to airway protection and bolus transit; its dysfunction may lead to aspiration or respiratory compromise [8,9]. It also interacts with mandibular and cervical musculature, playing a stabilizing role in head posture [8,11]. The Functional Masticatory Angle of Planas (AFMP) provides a clinical tool to assess masticatory symmetry; deviations in AFMP may reflect unilateral function and associated neuromuscular asymmetry [6].

Despite the recognized importance of mastication and hyoid biomechanics, few studies in orthodontics have examined their direct relationship using clinically accessible tools. Most existing evidence comes from imaging techniques such as CBCT or ultrasound, which, while accurate, are costly and not feasible for routine clinical use [3,10]. The AFMP therefore represents a simple, radiation-free parameter to evaluate masticatory symmetry, yet its association with hyoid bone position remains unexplored in clinical research. This pilot study aims to bridge this gap by assessing the relationship between AFMP and hyoid position, contributing preliminary data to guide future large-scale and longitudinal studies.

## 2. Materials and Methods

Study design. A descriptive, observational, cross-sectional pilot study was conducted to explore a potential association between masticatory patterns and hyoid bone position. The sample was divided into two groups: one with aligned hyoid position on both sides, and another with asymmetrical hyoid positioning. This design was chosen as an exploratory pilot to detect potential trends before conducting larger longitudinal studies. The classification into aligned vs. asymmetrical hyoid groups followed a predefined radiographic criterion based on vertical level differences between the right and left hyoid branches

Study population. Participants included orthodontic postgraduate students, dental students, university clinic staff, and acquaintances of the research team. All were evaluated at the university’s clinical facilities. Participants were recruited consecutively from the UCV university clinics during January–March 2025.

Inclusion and exclusion criteria. Participants were eligible for inclusion if they were healthy males or females between 12 and 31 years of age, had no history of orthodontic treatment or neurological conditions, and were undergoing orthodontic assessment at the Universidad Católica de Valencia (UCV). For participants under the age of 18, written informed consent was obtained from a legal guardian in addition to the participant’s assent.

All participants underwent a preliminary orthodontic screening to verify eligibility, which included the assessment of occlusion, dental integrity, and general health status. The screening also confirmed the absence of prior orthodontic treatment, neurological conditions, or balance disorders. Exclusion criteria were a history of orthodontic treatment, previous surgeries involving the oral cavity or lower limbs, edentulism or the absence of more than six teeth, craniofacial syndromes or marked facial asymmetries, balance disorders, recent episodes of vertigo, pregnancy, or failure to meet the age range of 12–31 years.

Sample Size. No formal sample size calculation was conducted, as this was an exploratory pilot study. However, based on methodological recommendations for pilot designs, a sample of 18 participants was deemed sufficient to assess feasibility, intraobserver reliability, and variability of measurements for planning future studies.

Data collection and instrumentation. Masticatory type was assessed using the Functional Masticatory Angle of Planas (AFMP) from standardized intraoral photographs, while hyoid bone position was evaluated on panoramic radiographs (orthopantomograms) obtained with the Dentsply Sirona X-Mind Pano D+ unit.

All intraoral photographs were taken by the same trained orthodontic postgraduate operator using standardized cheek retractors to ensure full visibility of occlusal planes. A Canon 250D camera with a 100 mm Sigma macro lens was used. Images were saved in JPG format, imported into Microsoft PowerPoint, and magnified at a uniform scale before tracing reference axes. AFMP angles were then measured by overlaying a digital protractor (PNG format) on the traced axes to minimize measurement error.

Panoramic radiographs were acquired following manufacturer positioning guides and standardized patient instructions (upright posture, feet together, tongue against the palate, lips closed) to minimize image distortion. Each image was reviewed for positioning errors before measurement.

**Figure 1:**
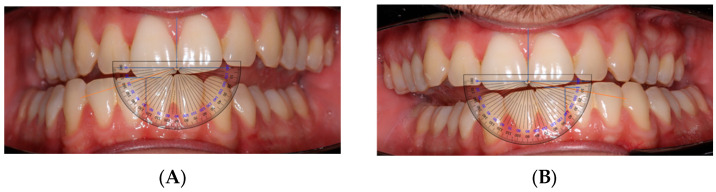
(**A**): Right-side AFMP measured using a protractor overlay. (**B**): Left-side AFMP measured using a protractor overlay.

**Figure 2:**
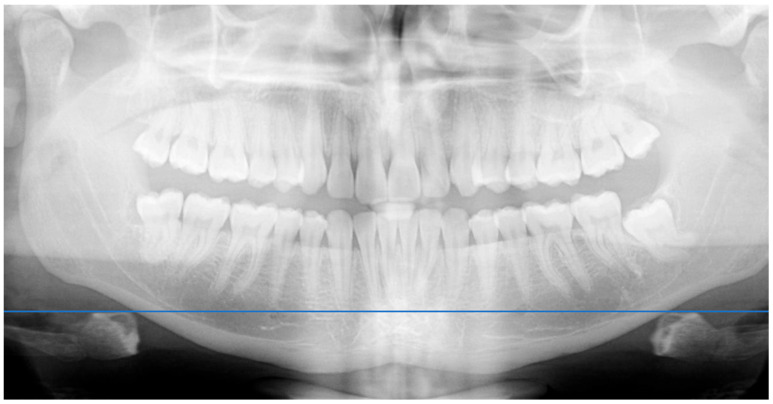
Horizontal reference line drawn for comparative analysis of hyoid bone position.

Clinical protocol. All procedures took place at UCV university clinics. After signing informed consent, participants underwent:

1. Photographic Records: Three intraoral photos were taken per patient—one in maximal intercuspation (MI), and two during mandibular lateralization to the right and left. Patients and operator were seated at equal height to standardize angulation of images.
2. AFMP Measurement: Horizontal and vertical reference axes were traced on images using PowerPoint. Angles were then measured using a transparent angle protractor overlay (Figure 1). Reference axes were defined using the maxillary incisal edge line and the mandibular interincisal point; vertical axes corresponded to the dental midlines. This procedure was selected as a pragmatic, low-cost alternative to specialized software. It should be regarded as a preliminary clinical tool rather than a standardized protocol.
3. Radiographic Analysis: A panoramic radiograph was obtained for each participant. A horizontal line was drawn across the hyoid bone, and vertical distances were measured from the upper border of the hyoid to the antegonial notch on each side using a digital ruler overlay (Figure 2). Distances were measured digitally from the most superior point of the hyoid body to the antegonial notch bilaterally, using an on-screen ruler calibrated at the same scale.

Image quality and positioning control. All radiographs followed standardized acquisition protocols. To minimize image distortion, patient posture, head alignment, and tongue position were carefully controlled. The operator followed manufacturer recommendations and ensured alignment with anatomical planes (sagittal and Frankfort). Proper positioning was critical to avoid magnification errors, especially in younger patients [14,15,16]. Operator experience and patient cooperation were emphasized, as involuntary movements or incorrect tongue position are known to produce magnification and ghost-image artifacts in panoramic radiography.

Statistical analysis. Descriptive statistics were generated. An ANOVA test was applied to evaluate associations between AFMP differences and hyoid position. To assess intraobserver reliability, a second round of measurements was performed in 10 randomly selected patients after 30 days. Intraobserver reliability was assessed not only by repeating measurements but also by calculating the Standard Error of Measurement (SEM) and Intraclass Correlation Coefficient (ICC, Shrout and Fleiss model), which were interpreted according to accepted thresholds (>0.9).

Ethical Considerations. Participation required informed consent. All participants were anonymized using numerical codes, and data confidentiality was preserved according to current data protection regulations. The study was approved by the UCV Ethics Committee under code UCV/2024-2025/009.

## 3. Results

The sample selected for this descriptive study consisted of a total of 18 participants. The mean age of participants was 21.4 years (SD = 5.2; range: 12–31), with 7 males and 11 females. Each individual underwent an evaluation of the position of the hyoid bone through 2D panoramic radiography, in relation to their AFMP, assessed individually. The analysis of both parameters suggests a potential imbalance in overall body posture.

**Figure 3:**
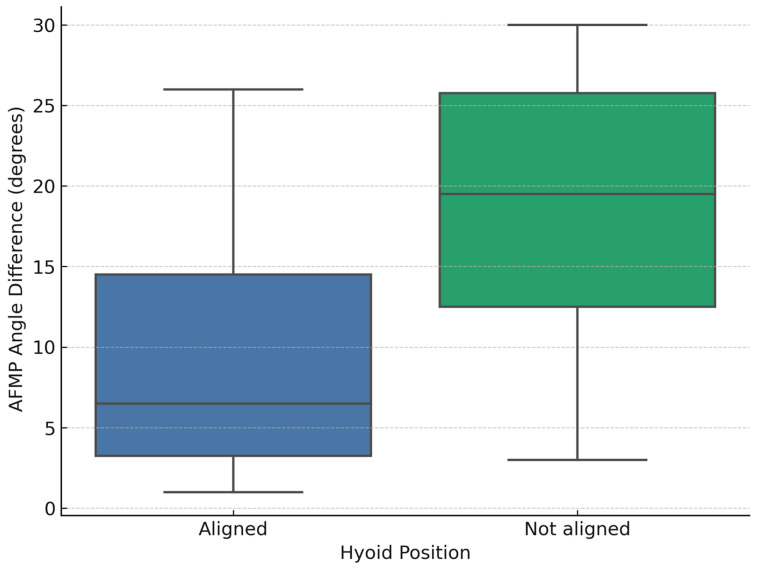
Distribution of AFMP angle differences according to hyoid bone position. Boxplot showing AFMP angle differences (*y*-axis, in degrees) by hyoid position (*x*-axis). Blue = aligned (hyoid at the same level); Green = not aligned (unilateral elevation).

To facilitate the analysis, the sample was divided into two groups according to the radiographic symmetry of the hyoid bone. The first group included participants whose hyoid bone appeared aligned on both the right and left sides in the panoramic radiograph. The second group comprised those in whom the hyoid presented an asymmetrical position, characterized by a unilateral elevation. Out of the 18 participants, 10 exhibited an aligned hyoid, while the remaining 8 showed a visible asymmetry (Figure 3). In the aligned group, the mean absolute AFMP difference was 9.3° (SD = 8.2°; range: 1–26°). In contrast, the non-aligned group showed a mean absolute AFMP difference of 18.6° (SD = 9.5°; range: 3–30°). Raw AFMP values for each participant are provided in Supplementary Table S1 to facilitate reproducibility.

**Figure 4:**
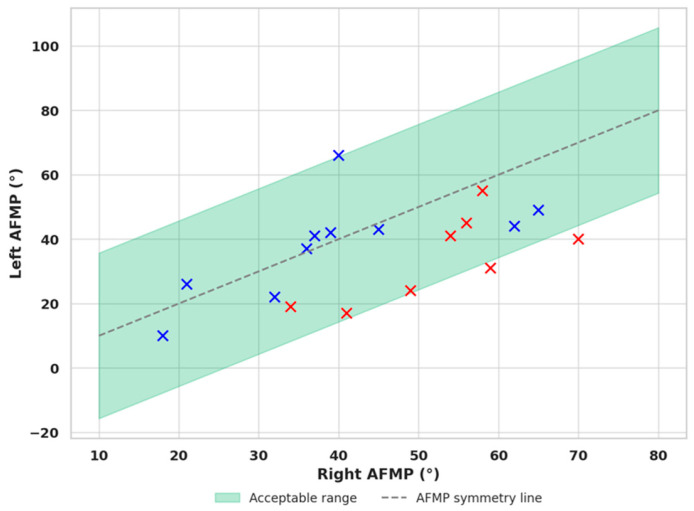
Scatter plot of right versus left AFMP values (in degrees). Each point represents one participant; blue = aligned, red = displaced. The diagonal indicates symmetry, with greater deviations reflecting higher functional asymmetry.

When plotting the right and left AFMP values on a scatter plot, participants were categorized into two groups based on hyoid bone position: those with an aligned hyoid (blue markers) and those with a displaced hyoid (red markers). This classification enabled the construction of a distribution graph to visualize the relationship between AFMP asymmetry and the vertical displacement of the hyoid bone (Figure 4).

The calculated mean of the absolute difference between right and left AFMP values in the aligned hyoid group was 9.3°, with a standard deviation of 8.2°. Based on this, preliminary variability range between 0° and approximately 25.7° was observed in this sample, although this upper limit may be overly permissive and should be interpreted with caution until confirmed in larger cohorts. When observing the scatter plot, most blue markers (aligned hyoid cases) clustered near the line of AFMP symmetry. Only three cases deviated substantially, warranting closer clinical evaluation. For instance, a patient with a lifelong left-dominant masticatory pattern may have recently undergone a dental restoration that altered their lateral function to the right. Such a shift may not yet be reflected in the hyoid position but could affect AFMP measurements, leading to misclassification. A larger sample size and detailed clinical histories are needed to identify and exclude patients with recent or transitional masticatory changes.

A one-way ANOVA was conducted to assess the relationship between AFMP asymmetry and hyoid bone position. The results indicated a significant effect, F(1, 16) = 4.97, *p* = 0.040, with an effect size of η^2^ = 0.24, suggesting a moderate-to-strong association. The 95% confidence interval for the mean AFMP difference between groups was [0.2°, 18.4°], confirming a moderate-to-strong effect size.

Intraobserver reliability analysis for AFMP measurement. To evaluate the intraobserver reliability of the AFMP measurements, a second assessment was conducted 30 days after the initial evaluation in a randomly selected subgroup of 10 participants. The mean difference between the two measurements was 0.9° on the right side and −0.2° on the left, with standard deviations of 2.28 and 1.99, respectively. The corresponding standard errors of measurement (SEM) were low—1.61° on the right and 1.41° on the left—indicating minimal variation across repeated assessments. These findings suggest a high degree of measurement consistency. The ICC, calculated using the Shrout and Fleiss model for absolute agreement, were 0.984 for the right side and 0.990 for the left, both of which fall within the range considered to represent excellent reliability. The SEM was 1.61° (right) and 1.41° (left), supporting the precision of repeated measurements. Collectively, these results support the reproducibility and clinical validity of the AFMP measurement protocol employed in this study.

**Intraobserver reliability in the measurement of hyoid bone position on panoramic radiographs.** To determine the intraobserver reliability of hyoid bone position measurements, a second set of assessments was conducted 30 days after the initial measurements in a randomly selected subgroup of 10 patients. The evaluation involved quantifying the vertical distance from the most superior point of the hyoid bone to the antegonial notch on both the right and left sides using standardized reference points on orthopantomographic images. Remarkably, all repeated measurements were identical to the initial values across all patients, yielding a mean difference of 0.0 mm, a standard deviation of 0.0 mm, and a standard error of measurement (SEM) of 0.0 mm for both sides. Both SEM and SD of the repeated hyoid measurements were 0.0 mm, indicating perfect reproducibility of the method.

The absence of any variability between the two measurement sessions indicates exceptional consistency in the applied methodology. To further substantiate these findings, the ICC was calculated using the Shrout and Fleiss model for absolute agreement in a two-way random-effects design. The ICC values for both the right and left sides were 1.000, representing the highest level of statistical reliability achievable and confirming perfect agreement between the two sessions. However, this perfect value resulted from identical raw measurements and should be interpreted with caution, as such absolute indices are uncommon in clinical research and may partly reflect rounding or methodological constraints.

These results provide strong evidence of the methodological robustness and reproducibility of the radiographic protocol employed in this study. The use of clearly defined anatomical landmarks and a standardized digital overlay for distance measurement, combined with consistent image acquisition parameters, likely contributed to the observed precision. Taken together, the findings confirm that the technique used to evaluate hyoid bone position on 2D panoramic radiographs is not only replicable but also reliable for future clinical and research applications.

## 4. Discussion

While interpreting the findings of this study, it is important to recognize certain methodological considerations that may influence the conclusions drawn. One of the main challenges lies in the current lack of a universally accepted reference standard to definitively establish whether hyoid bone position serves as a reliable marker of masticatory pattern. Despite this, the results gain strength from consistent biomechanical foundations: sustained activity of perioral musculature on the dominant chewing side may induce adaptive muscular shortening, potentially leading to an upward displacement of the hyoid bone on the same side—an outcome that is detectable through panoramic imaging.

From a physiological and anatomical standpoint, previous studies suggest that the hyoid bone does not maintain a fixed position but rather adapts to various anatomical and functional factors [17]. The bone’s position and development are closely tied to muscular activity, and when exposed to uneven masticatory loads, the resulting asymmetry may impact skeletal remodeling [18,19]. If one side experiences greater muscle activation, trabecular patterns in the associated bone may become denser, while inactivity may lead to displacement or atrophy [18]. Muscle insertions exert a direct influence on bone development, positioning skeletal structures as reactive elements to muscular stimuli [18]. Repetitive functional loads may induce muscular hypertrophy or adaptive changes based on the physiological mechanisms of contraction, regulated by sarcomeric elements such as actin and myosin [20,21]. These adaptations are highly relevant in the context of mastication.

Unilateral functional overload could disrupt the equilibrium of the stomatognathic system, prompting adaptive muscular responses that explain the unilateral elevation of the hyoid observed in patients with dominant masticatory patterns [17,18]. The consistent elevation of the hyoid bone on the side corresponding to the smaller AFMP in our sample aligns with this hypothesis, supporting a model in which repeated asymmetrical muscle activity influences hyoid positioning. Although skeletal characteristics were not the focus of this study, previous research has indicated that craniofacial growth patterns and malocclusions are also associated with hyoid positioning [17], suggesting potential directions for future investigation. Therefore, these findings should be interpreted with caution, as they are exploratory and not definitive evidence.

Alternative methods such as EMG and CBCT provide detailed information on muscle activity and skeletal asymmetries but require specialized equipment, higher cost, and longer analysis. In contrast, AFMP offers a simple, low-cost, radiation-free measurement from intraoral photographs, making it uniquely advantageous for routine clinical workflows.

One of the most evident limitations of this study is the small sample size, consisting of only just 18 participants. As a pilot study, the primary aim was to detect trends rather than draw definitive conclusions. However, future studies with larger cohorts will be essential to confirm these preliminary associations and to evaluate whether normalization of masticatory function might restore hyoid symmetry. In addition, there is currently no universally accepted protocol for evaluating hyoid position on panoramic radiographs. Although intraobserver reliability in this study was excellent, the use of a self-developed measurement method introduces a potential bias and should be validated by independent examiners. In this context, AFMP measurements were performed using PowerPoint with a transparent protractor. This pragmatic, low-cost method allowed consistent and reproducible assessments, supporting its clinical applicability. However, as it is not an established standard, it must be considered a preliminary approach and acknowledged as a limitation pending validation in future studies. Likewise, the perfect ICC values obtained (1.000) were a consequence of identical repeated measurements. While they indicate excellent reproducibility, such absolute values are atypical in clinical research and may partly reflect rounding or methodological constraints. They should therefore be interpreted with caution and regarded as a methodological limitation rather than conclusive evidence of absolute reliability.

Another relevant limitation involves the use of 2D radiographs, which are susceptible to magnification errors, distortion, and spatial ambiguity regarding the true anatomical position of the hyoid. Although standardized positioning protocols were applied to minimize these effects, such errors are inherent to 2D imaging and must be taken into account when interpreting the results. A more accurate assessment would involve three-dimensional imaging, such as CBCT, to validate the findings. Complementary functional tests could also enhance the understanding of preferred masticatory patterns. Techniques like electromyography could offer objective data on muscle activity and asymmetry, while podiatric assessments using pressure platforms may reveal postural compensations linked to unilateral mastication.

As with any cross-sectional design, this study is limited by its inability to capture longitudinal changes or establish causality between masticatory patterns and skeletal adaptations such as hyoid displacement. However, the findings reveal consistent tendencies that support further exploration. Notably, all cases of hyoid elevation observed on the left side corresponded with a reduced AFMP on that same side, reinforcing the hypothesis that decreased masticatory angle amplitude may be associated with dominant functional use and result in measurable anatomical consequences. Conversely, individuals with aligned hyoid positions exhibited a wider range of bilateral AFMP differences. While most values remained near the axis of symmetry, a few showed substantial discrepancies, possibly reflecting transitional masticatory behaviors or recent occlusal modifications. These findings highlight the need for larger sample sizes and more detailed clinical histories to better define acceptable ranges of AFMP variability and to distinguish between stable patterns and recent functional shifts. Future research should aim to include a greater number of cases with right-sided hyoid elevation to ensure subgroup balance, integrate complementary clinical variables such as postural and podiatric parameters, and employ longitudinal designs to evaluate whether masticatory rehabilitation or orthodontic treatment can influence hyoid positioning over time.

Furthermore, potential confounding factors such as parafunctional habits (e.g., bruxism or clenching) and postural patterns (e.g., forward head posture or cervical compensations) were not controlled in this study and could also have influenced both hyoid position and masticatory dynamics. Future research should take these variables into account to obtain a more robust interpretation of the observed associations.

## 5. Conclusions

This pilot study exploratorily suggests a potential relationship between hyoid bone position and masticatory pattern, as an elevated hyoid was frequently observed on the same side as the smaller AFMP. Although some cases with symmetrical hyoid positioning showed notable AFMP differences, the overall pattern may support the hypothesis of functional asymmetry influencing anatomical adaptation. The use of AFMP measurement appears to be a potentially useful tool for assessing masticatory dynamics in relation to craniofacial structure. While these findings are not statistically conclusive, they should be considered hypothesis-generating and underscore the need for further research with larger cohorts to clarify the clinical significance of the observed associations.

## Data Availability

The original contributions presented in the study are included in the article and Supplementary Materials, further inquiries can be directed to the corresponding author.

## Supplementary Materials

The following supporting information can be downloaded at: https://www.mdpi.com/article/10.3390/dj13100451/s1. Table S1: Raw AFMP values and hyoid bone position for each participant.
