# Supplementary material for: Functional Masticatory Angle and Hyoid Bone Position: A Pilot Study on Occlusal Symmetry and Morphofunctional Adaptation
Source: Dent J (Basel). 2025 Oct 1;13(10):451. doi: 10.3390/dj13100451 (PMC12563113; doi:10.3390/dj13100451)
Supplement: Supplementary file 1 [file dentistry-13-00451-s001.zip › dentistry-3857765-supplementary.pdf]

dentistry journal

Article

Functional Masticatory Angle and Hyoid Bone Position: A Pilot Study on Occlusal Symmetry and Morphofunctional Adaptation

Lorena Sigwald-Serpa [1], Icíar Sanz-Orrio Soler [1], Laura Marqués-Martínez [1,*], Juan-Ignacio Aura-Tormos [2], Esther García-Miralles [2] and Clara Guinot-Barona [1]

Supplementary Table S1: Raw AFMP values and hyoid bone position for each participant.

Patient	Right AFMP (°)	Left AFMP (°)	Absolute Difference (°)	Hyoid Position
P1	58	55	3	Left elevated
P2	36	37	1	Aligned
P3	59	31	28	Left elevated
P4	56	45	11	Left elevated
P5	41	17	24	Left elevated
P6	18	10	8	Aligned
P7	70	40	30	Left elevated
P8	40	66	26	Aligned
P9	49	24	25	Left elevated
P10	37	41	4	Aligned
P11	45	43	2	Aligned
P12	65	49	16	Aligned
P13	34	19	15	Left elevated
P14	54	41	13	Left elevated
P15	39	42	3	Aligned
P16	62	44	18	Aligned
P17	21	26	5	Aligned
P18	32	22	10	Aligned

 https://doi.org/10.3390/dj13100451
